# Australian Patient Preferences for the Introduction of Spirituality into their Healthcare Journey: A Mixed Methods Study

**DOI:** 10.1007/s10943-022-01616-3

**Published:** 2022-08-03

**Authors:** Megan C. Best, Kate Jones, Frankie Merritt, Michael Casey, Sandra Lynch, John Eisman, Jeffrey Cohen, Darryl Mackie, Kirsty Beilharz, Matthew Kearney

**Affiliations:** 1 Institute for Ethics and Society, University of Notre Dame Australia, Broadway, PO Box 944, Sydney, NSW 2007 Australia; 2 The School of Medicine, The University of Notre Dame, Sydney, Australia; 3 Garvan Institute of Medical Research, Sydney, Australia; 4 St Vincent’s Private Hospital, Sydney, Australia; 5 Excelsia College, Sydney, Australia; 6 St Vincent’s Hospital, Sydney, Australia

**Keywords:** Spirituality, History-taking, Patient preferences, Holistic care, Healthcare professionals

## Abstract

While patients value engagement concerning their spirituality as a part of holistic healthcare, there is little evidence regarding the preferred way to engage in discussions about spirituality. This study investigated inpatient preferences regarding how they would like spirituality to be raised in the hospital setting. A cross-sectional survey was conducted with inpatients at six hospitals in Sydney, Australia (*n* = 897), with a subset invited to participate in qualitative interviews (*n* = 41). There was high approval for all proposed spiritual history prompts (94.0–99.8%). In interviews, the context dictated the appropriateness of discussions. Findings indicated a high level of patient acceptability for discussing spirituality in healthcare. Further research and more detailed analysis is required and proposed to be undertaken.

## Introduction

Spirituality in healthcare refers to a dynamic and intrinsic aspect of humanity through which individuals seek ultimate meaning, purpose and transcendence, and experience their relationships to family, others, community, society, nature and the significant/sacred. Spirituality is expressed through beliefs, values, traditions and practices or rituals (Puchalski et al., 2014). One way to characterise spirituality is through relationships. The common relationships identified are those with family, a higher being or the transcendent, with nature or culture, or with the person’s inner sense of self (Sulmasy, 2002).

Research has demonstrated the health benefits of high levels of spiritual well-being. These include pain relief and reduced suffering, improved adjustment to illness and disability, prevention and improved outcomes in diseases including depression, heart disease and cancer (Abu et al., 2018; Braam & Koenig, 2019; Hilbers et al., 2010; Jones et al., 2019, 2020; Koenig et al., 2012; Siddall et al., 2017). When patients are asked about their spiritual beliefs, it improves healthcare relationships, increasing trust, and giving the patient confidence to ask difficult questions, such as how long s/he has to live (Best et al., 2014). Spiritual care will therefore be an important part of achieving holistic person-centred care.

The importance of spiritual care has been recognised at a policy level in Australia, with national organisations mandating accessibility of safe and high-quality spiritual care in healthcare and aged care in view of its importance to quality of life and well-being (Meaningful Ageing Australia, 2016; Spiritual Health Association, 2020). It is important to know how clinicians can most effectively raise the topic with patients.

While there is widespread agreement that questions about spirituality are beneficial in the healthcare space (Best et al., 2015c), with high patient acceptance (Best et al., 2015b), there is little evidence regarding the preferred way to engage in spirituality discussions with the Australian public, and spiritual history-taking is still not routine practice (Rombola, 2019). Commonly used spiritual history taking tools have been developed in the USA and the UK (Anandarajah & Hight, 2001; Borneman et al., 2010; Maugans, 1996; Neely & Minford, 2009; Ross & McSherry, 2018), and it cannot be presumed that models of history-taking transfer across different cultures (Best et al., 2020, 2022; Jones et al., 2021; Rombola, 2019). Furthermore, much of the literature about spirituality in healthcare has focussed on the end of life setting, despite the knowledge that spiritual care is relevant in a wide variety of health contexts (Appleby et al., 2018).

We aimed to investigate the most acceptable person-centred way to assess spirituality and spiritual care needs in Australian hospitals, with the aim to produce a culturally sensitive history-taking tool for Australian healthcare, that is, a history-taking tool that is aware of Australian cultural attitudes. This information is needed for several reasons. Firstly, given the importance of spiritual well-being, it is essential to equip Australian health professionals with tools that enable them to collect relevant information and provide appropriate care effectively and sensitively. There is evidence that even asking about spirituality was interpreted by patients as a form of spiritual care and this provides support for routine screening questions to be used with all patients (Best et al., 2015a; Chochinov & Cann, 2005); however Rombola and colleagues found that lack of confidence to take a spiritual history was a barrier for 79% of Australian doctors in their study (Rombola, 2019).

Secondly, general healthcare staff are not trained to manage spiritual crises, and, in times of financial limitations, knowledge of patients' needs will inform where specialist spiritual care services such as pastoral care (also known as spiritual care or chaplaincy) are focused (VandeCreek, 2001). Thirdly, given competence in spiritual history-taking is associated with reduced levels of burnout in the health professions (Girgis et al., 2009), inclusion of spiritual history-taking in healthcare would also contribute to staff well-being.

This paper describes a study which aimed to explore the most effective way to take a spiritual history from Australian patients. In particular we aimed to identify the following:

1. The preferred wording for discussing spirituality in a hospital setting
2. Demographic features of patients in relation to their preferences regarding spiritual history-taking.

We also asked participants which staff member was preferred for these discussions, and this data will be reported in a future paper.

## Methods

### Research Design

This is a mixed-method cross-sectional study comprised of a short survey and semi-structured qualitative interviews.

## Participants

Participants were recruited from six hospitals across Sydney, Australia. Hospitals included public and private facilities, comprising both acute and sub-acute inpatient as well as outpatient care, and represented a combined total of over 1,000 beds. Hospitals included both faith-based and non-faith-based institutions. Eligible patients were adult; alert, oriented and able to give verbal consent; able to understand and speak English; and well enough to participate in the study.

Eligible patients were identified by nursing unit managers at the participation sites and approached by a researcher, who asked whether they were willing to participate in a short survey, explained what it involved and answered any questions. Verbal consent was obtained before the survey was distributed and documented by the return of an anonymous survey (implied consent). Researchers were trained to assist with administration of the survey in a non-coercive way if required (QOL Office, 2019).

The survey contained an invitation to participate in a qualitative interview to explain survey responses. If the patient expressed interest in participating in an interview, information about the process was given and the opportunity to ask questions provided. After written consent was received, patient contact details were recorded in order to arrange an interview at a convenient time, according to the sampling protocol. Ethics approval was granted by the Human Research Ethics Committee at St Vincent’s Hospital, Sydney (HREA AU/1/B78D25).

## Procedure

All participants were asked to complete a survey which included the following:

*Demographic details:* age, sex, education level and main lifetime occupation (proxy for socioeconomic level), indigenous status, religion, and length of stay (proxy for severity of illness).

*Preferred spiritual history questions:* Patients were asked to consider alternative prompts about their spirituality based on documented spiritual history-taking tools. These tools were chosen as they were known to be currently in use in the local context. They were asked whether it was acceptable to be asked to respond to the prompts in a healthcare context (with the option of recording inability to understand the prompt). The three history tools were the following:

1. A list of questions used by experienced palliative care physicians when eliciting a spiritual history in the Australian and New Zealand context, as identified in a study by Best et al. (Best et al., 2015a). These were: What’s really important to you when times are tough? What or who is the most important thing in your life?  What or who keeps you strong when times are tough?
2. The FICA© spiritual history tool (Puchalski & Romer, 2000), is a guide for conversations about spirituality in the clinical setting developed in the USA, and comprised of four question areas about: (1) Faith and belief; (2) Importance (of faith or belief); (3) Community (religious or spiritual); and (4) Address in care (within the healthcare setting).
3. A single clinician-administered item ‘Are you at peace?’ validated in American patients with advanced serious illness as a measure of spiritual well-being (KE Steinhauser et al., 2006) using a 5-point Likert scale.

*Self-assessment of spirituality and religiosity:* The Multidimensional measurement of religiousness/spirituality for use in health research comprises two items asking whether individuals considered themselves spiritual or religious with a 5-point Likert scale (Fetzer Institute and National Institute on Aging, 2003).

A subset of patients was invited to complete a qualitative interview. Purposive sampling was used to ensure heterogeneity in the sample. Those who agreed to participate were either interviewed in the ward or scheduled for a telephone interview within 1–2 weeks of giving consent. Interviews were conducted by two researchers (KB and KJ) and continued until data saturation. Interviewers asked patients about their attitudes to being questioned by healthcare staff, about their spiritual beliefs and practices and to explain the rationale for the answers they gave in the survey.

## Analysis

*Quantitative:* Demographic data were tabulated, and descriptive statistics generated to describe the results. A series of Fisher’s Exact tests were conducted to investigate differences between categorical variables on patient preferences for each question. Associations with sex, age, patient diagnosis, religious affiliation, and self-identified spirituality and religiosity were examined. Effect sizes were measured using Cramer’s V and considered to be “small” if < 0.3.

*Qualitative:* Interviews were recorded and transcribed verbatim by a professional transcription company. Free text responses to the questionnaires were added to the transcripts for analysis. Theoretical thematic analysis (Braun & Clarke, 2006) was used to code qualitative data.

After familiarising themselves with the data, three researchers (MB, KJ and FM) manually coded six manuscripts to form initial codes. These preliminary codes were then used to synthesise groups of data into focussed codes which were applied to a further six transcripts to establish agreement on coding and refine the code tree, which was then applied to the remaining transcripts. New codes were developed iteratively as required and were collated to develop themes. Themes were developed and discussed by the research team, which comprised experts in medicine and allied health.

Differences were resolved through discussion and negotiated consensus, therefore allowing reflection on the role of our individual perspectives in the interpretation of data. Rigour was derived from successive rounds of discussion and development of focused codes, definitions and themes and review of the coding process by all authors until theoretical coding was complete. Theoretical sampling allowed exploration of each code until they were well understood. Illustrative quotes for each theme were extracted from the transcripts. Triangulation of data was achieved through comparison of qualitative and quantitative results.

## Results

### Quantitative

There were 897 patients who completed the survey. Approximately half were female, with a diverse age range. Four hundred and seventy-five (52.9%) patients considered themselves ‘spiritual’ (agree or strongly agree), while 345 (38.4%) considered themselves ‘religious’. The largest religious group was Protestant (36%), followed by Roman Catholic/Orthodox (28%) with no religion accounting for 26%, reflecting proportionally a slightly more religious sample than the last Australian census (30.1% No Religion in 2016). One third considered themselves to be neither spiritual nor religious. Patients were admitted to a wide range of hospital wards. Full demographic details are found in Table 1.

**Table 1:** Demographic details *n *= 897

Demographic items	Category	*N* (%)
Sex	Female	422 (47.0)
	Male	469 (52.3)
	Missing	6 (0.7)
Age (*n*, %)	20–29	60 (6.7)
	30–39	57 (6.4)
	40–49	62 (6.9)
	50–59	115 (12.8)
	60–69	173 (19.3)
	70–79	224 (25.0)
	80 and over	126 (14.0)
	Missing	80 (8.9)
Patient diagnosis (*n*, %)	Medical	338 (37.9)
	Rehabilitation	84 (9.4)
	Palliative care	55 (6.1)
	Surgical	232 (25.9)
	Emergency Medicine	64 (7.1)
	Geriatric/Aged Care	35 (3.9)
	Psychiatry	10 (1.1)
	ICU	21 (2.3)
	Other	21 (2.3)
	Maternity	32 (3.6)
	Missing	5 (0.6)
Religious affiliation (*n*, %)	Protestant	326 (36.3)
	Catholic/Orthodox	254 (28.3)
	None (Atheist/Agnostic/None)	237 (26.4)
	Jewish	35 (3.9)
	Other religions*	39 (4.4)
	Missing	6 (0.7)
I am a spiritual person	Strongly disagree	31 (3.5)
	Disagree	271 (30.2)
	Neither agree nor disagree	99 (11.0)
	Agree	283 (31.5)
	Strongly agree	192 (21.4)
	Missing	21 (2.3)
I am a religious person	Strongly disagree	98 (10.9)
	Disagree	374 (41.7)
	Neither agree nor disagree	57 (6.4)
	Agree	167 (18.6)
	Strongly agree	178 (19.8)
	Missing	23 (2.6)
I am a religious or spiritual person	Not spiritual or religious	269 (30.0)
	Spiritual but not religious	147 (16.4)
	Spiritual and religious	322 (35.9)
	Neither agree nor disagree	40 (4.5)
	Religious but not spiritual	21 (2.3)
	Missing	98 (10.9)

*Other religions included Buddhism, Islam, Hinduism, and Indigenous spirituality

The majority of respondents were happy to be asked to respond to all prompts proposed as ways of beginning a discussion about  spirituality, with positive response rates ranging from 94.0 to 99.8% depending on the question. Fisher’s exact test identified a significant preference for the conversation prompt ‘I know what keeps me strong when times are tough’ by participants who self-identified as a religious person (*p* < 0.05). The prompt ‘I am at peace’ was significantly preferred by those who had a religious affiliation, and those who self-identified as a spiritual or religious person (*p* < 0.5). Due to the homogeneity of the responses, effect sizes of other associations were small and excluded from the analysis. There were no significant associations with Patient Diagnosis category. Results are summarised in Table 2.

**Table 2:** How would you feel if someone asked you to respond to this statement?

Statement	Happy to be asked *N* (%)
I consider myself spiritual	886 (98.8%)
I consider myself religious	886 (98.8)
I know what gives my life meaning	886 (98.8)
Faith or belief is important in my life	886 (98.8)
My beliefs influence how I handle stress	885 (98.7)
I am part of a spiritual or religious community	884 (98.6)
I have a community that is of support to me	843 (94.0)
My beliefs influence my healthcare decision-making	850 (94.8)
There is a group of people who are important to me	895 (99.8)
I know what’s really important to me when times are tough	893 (99.6)
I know what or who is the most important thing in my life	893 (99.6)
I know what or who keeps me strong when times are tough	852 (95.0)*
I know what gives me peace	884 (98.6)
I am at peace	850 (94.8)*

* indicates significant group differences (*p* < 0.05)

### Qualitative

**Fig. 1:**
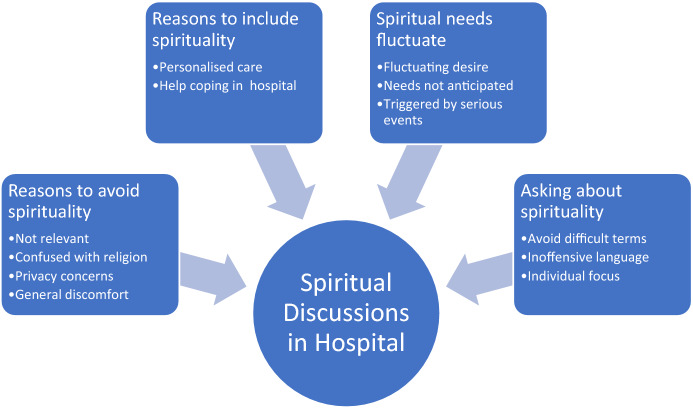
Australian patients’ preferences for spiritual discussion–themes and subthemes

Forty-one participants were interviewed. Participants expressed a wide range of views regarding how they would like to be asked about spirituality and initial reasons for discussion were focussed on the desire for personalised care rather than spiritual support. While some participants expressed hesitation to discuss spirituality in a healthcare context, there was general appreciation that the relevance of conversations depended on the context, with participants identifying triggers for discussion. Four themes were identified: (1) Reasons to avoid spirituality; (2) Reasons to include spirituality; (3) Spiritual needs fluctuate; (4) Asking about spirituality. See Fig. 1.

#### (1) Reasons to Avoid Spirituality.

Suggested prompts about spirituality were not offensive to most participants, as reflected in the quantitative results. Reasons proffered explaining why some patients might hesitate to engage in spiritual discussions with healthcare staff were: believing that spirituality was not relevant to healthcare; privacy concerns; and discomfort with the topic generally.

- ***Spirituality is Not Relevant to Healthcare***
  Some respondents saw healthcare as strictly a physical exercise, and as such they wanted the staff to focus on physical issues only. *'I’m here for another reason. I'm here because I'm crook’. (22) ‘It’s not so much privacy as relevance. You only need the information that’s going to help you provide my care’. (K3).*
  In part this was because spiritual issues were not seen as relevant to healthcare decision-making: *‘I’m 100% for people’s beliefs and their own religion and everything, but in my eyes, I can’t let my culture blind my rationality and logic with my health’. (11) ‘I wouldn’t necessarily be making decisions based on a Presbyterian view of something just because I’m a Presbyterian’. (25).*
  These comments often reflected a confusion between spirituality and religion, and a rejection of spirituality because of a previous negative religious experience: *‘Scandals in the Catholic church [are] a turn-off.’ (2);* and/or fear of proselytising: *‘We live in an age where there are more who are atheists than Christians. To force any kind of Christianity or impose any kind of structure on them is … rude’. (K1)* The very small minority of participants who did not want any discussion on the topic gave these kinds of explanations.
  Following clarification, some participants qualified their initial responses, saying that, if their spiritual needs did become relevant to their experience, they did not object to spirituality then being addressed by healthcare staff: *‘I wouldn’t welcome decisions about things like that unless there was some particular relevance’. (26).*
- ***Privacy Concerns***
  Many participants were in shared rooms, with only a fabric curtain separating the beds. The view was often expressed that they did not feel they had sufficient privacy to engage in conversations on topics as sensitive as spirituality. This was not necessarily a blanket opposition to spiritual discussions, as much as a request for an appropriate setting. *‘Remember, we are talking about people who don’t necessarily have the luxury of a private room like I have at the moment [patient in isolation]. So, as much as we pull the curtains, there is no privacy and you even risk embarrassing them. None of that stuff should be done in a hospital bed where the next person hears their spiritual needs. All of that sort of stuff should be done in a private room where people can have their own thoughts and know that they are their own thoughts’. (K1).*
  Others felt that spiritual concerns were too private to share with general staff: *‘I probably wouldn’t open up to any of the staff because it goes in reports. It gets typed up and everybody knows. … I don’t need that to happen. I need to know that that’s private between the two people’. (32)* This was the case for some even if they felt spirituality was relevant to their healthcare decisions: *‘I feel I know how I would make decisions based on things I’ve been talking to you about but I don’t think I’d really want to fly it from the flag post’. (26).*
- ***General Discomfort Regarding Spirituality***

Some patients just found discussions about spirituality embarrassing, sometimes resorting to humour when asked questions. *‘I'm inclined to be a bit flippant when things like that come up. And I might throw in a bit of a joke or something like that. You know, that's my nature’. (21).*

Others thought they heralded bad news, and that such discussions were a way of talking about imminent death. As such they were not interested. *‘Well, “at peace” to me is someone dead’. (7).*

#### (2) Reasons to Include Spirituality

As reflected in the quantitative results, most participants were happy to discuss spirituality in the healthcare context. The main reasons for this related to the desire for personalised care and increased support for coping with hospitalisation.

- ***Desire for Personalised Care***
  Many participants saw discussion of their spirituality as a way to let the clinician know them better and thus be better placed to offer personalised, holistic care in line with their expressed values. *‘I think if they know [about my spirituality], it would be easier for everyone. If they know then they will be able to understand more. They would be able to care more. Then, I'm not afraid’. (13).*
- ***Help in Coping with Hospitalisation***

Patients were also aware that clinicians could help them access their source of spiritual support which was an important coping mechanism for hospitalisation. *‘I think having the right support and people to talk to about [spirituality] when you’re having a tough experience in your life-it helps you as well; it doesn't have to be someone spiritual. It could be just like a friend but yeah’. (17) ‘I was in ICU for a fortnight. I was in a coma for 10 days. When I came to, the staff seemed to have realised that family was extremely important because there was always …always someone with me. They-I think they saw how close we really are. And that no one was going to leave me on my own. So, yeah, they realised that family was extremely important… They were very accommodating of that’. [30].*

When staff were not aware of the importance of spiritual supports such as family members, it could make the inpatient experience more difficult: *‘If she [the nurse] had just asked me and said, what do you need? What-what is that thing that will help you and I would have said my family. But instead, she just assumed that they were making me worse’. (11).*

#### (3) Spiritual Needs Fluctuate

With regard to events which would make spiritual discussion ‘relevant’ to the admission, several triggers were identified, relating to the trajectory of illness. The fluctuating nature of spiritual needs and difficulty specifying them in advance were also identified.

- ***Spiritual Needs Fluctuate***
  Some patients, particularly those with serious or chronic conditions and/or long-term hospitalisation, talked about a fluctuating desire to discuss spirituality with hospital staff. *‘In the beginning stages like when you're quite sick and unwell there's a-definitely-it's-different. Because-you're probably a little bit more scared and a little bit worried about-how you're going to go, or how you're going to become better. Yeah, it definitely ebbs and flows. It's definitely dependent on where you're at. Like, vulnerability scale type thing’. (31) ‘I think the longer term the issues are, the more important [spirituality] becomes, you know? If you’re just going in the hospital for a few days, it’s kind of, not a huge issue, but when we start talking into weeks and months it can become quite important.’ (29).*
  Some patients with minor problems were also aware that this could happen: *‘I’m sure if the situation were more dire I would [want to be asked about spirituality]-I would think about it quite differently [from now] probably because I’m okay and I’m going to get out of here this week.’ (4)* It was also reported that interest in spirituality could grow in response to illness, as one patient who had suffered a spinal cord injury noted: ‘*It’s [spirituality] been there, but, yes, it has become more meaningful because you’ve got to try and have some way just to cope with this’. (32)* Many patients noted that their response to prompts such as whether they were at peace had changed since the period prior to their admission.
- ***Spiritual Needs May not be Known in Advance***
  Some participants mentioned that patients may not be aware that spiritual needs will arise prior to a crisis: *‘I don’t know if everybody knows … a lot of people think they have the resources to cope but they don’t. You don’t find out until you’re tested. People are often painfully unaware of their resilience or lack thereof’. (K3)* As needs could change over time, many patients recommended that staff regularly check whether a patient wanted to discuss spiritual issues: *‘Not every day but often. Yeah. Every couple of weeks, especially when it’s long term recovery, every couple of weeks. Just to get a sense of what’s changing for you. Yep. Yeah… It changes drastically’. (30).*
  For this reason, prompts about spirituality were encouraged that focussed on the need for support in the healthcare context, rather than general outlook on life: *‘Really just asking what kind of support you could offer them would be better than asking them about their perspective on how contented they are with life. I’m overall very contented with my life and I’m very lucky to have a good life, but that wouldn’t be reflected in those questions at the time I was sitting in pre-admission [when I was worried about my health situation].’ (K3).*
- ***Triggers for Spiritual Care***

Patients identified several factors, including the patient’s mood, which should make staff aware of the need to ask about spiritual needs. One participant related an incident where spiritual conversation had helped her enormously after a nurse noticed how despondent she had become: *‘I had an episode where I was really feeling really, really unwell and I just could-couldn’t feel as though I could fight and I thought, oh, I’ve had enough, I can’t do this anymore, that’s-that’s it, you know. So then I had a young-one of the young nurses, come in and he just said, “Come on, you’ve been through so much over the weeks and you’ve got through and, do you want-would you like to talk about how you’re feeling? …and they were just able to get me back on track just with a few words.” (14).*

Serious events such as impending death were also recognised as appropriate times to address spirituality: ‘*A lot of people with life threatening situations--don't know what to do. Don't know what to think. You know, they can be quite worried’. (13)* One patient who had been a midwife agreed: *‘I can tell you when, I did midwifery, and it’s very hard to cope with a mother that’s lost a baby’. (27).*

#### (4) Asking About Spirituality

As shown in the quantitative results, there was high acceptability for all prompts about spiritual topics for this cohort, and while the survey was not a good discriminator for preferred vocabulary, the qualitative responses gave more insight into which terms resonated for the patients interviewed. The term ‘spirituality’ was unfamiliar and/or uncomfortable for most participants. Most participants expressed the desire for a respectful approach, within a therapeutic relationship, with an individual focus, at the appropriate time.

- ***What is Spirituality?***
  The term ‘spirituality’ was unfamiliar for many patients, some of whom confused the term with ghosts (‘spirits’) or just were not sure what it meant: *‘[When asked ‘What does that word, spirituality mean to you?’] ‘Nothing much. I don’t think I’ve ever come across anybody that says it’. (27)* This response to being asked if spiritual discussions were acceptable, revealed the common confusion with religion noted above: *‘I think on a hospital form asking what religion you are is relevant because if you do die, you need to know who to call. So I think that’s OK’. (K3)* Participants often requested a definition of the term when asked to describe their views about spirituality.
  However, many participants were familiar with the ‘broad’ definition of spirituality, as a dimension of humanity and source of personal values: *‘[Spirituality is] where I think I can get support from, I suppose. Like, besides my family and friends, what my belief would give me [regarding] the support and encouragement I need’. (15) ‘When you say it [spirituality], my mind goes to religion. But it’s more than that. Spirituality is-my inner peace. It’s my-where I’m centred. I’m not a religious person’. (30)* For these reasons, asking up front about ‘spirituality’ was found to be a barrier for this cohort of Australian patients.
- ***Inoffensive Language***
  When asked how staff should frame questions about spirituality, some patients wanted straightforward questions rather than staff trying to work out the best way to ask: *‘I wouldn’t mind if they asked outright--but I wouldn’t care if they just waited and it came up naturally. So either-either way for me, I’m happy with [them], yeah’. (11)* Patients wanted their beliefs respected and not have the beliefs of others forced on them: *‘As long as no one’s trying to preach to me, I don’t mind what they ask me’. (K6) ‘It's understanding a person. It’s basically just putting aside your spiritual understanding and your beliefs, you understand the person for who they are, and then, you try and slowly incorporate spiritualism [sic] with that. That means you won't come off as offending that person’. (17).*
  A gentle approach was recommended when the patient was feeling vulnerable: ‘*If somebody kept saying to me, “Do you want to see somebody?” I’d say, “No, don’t ask me if I want to see-“ like I would feel that defensive- but it was very gentle. So in that way- that’s how I would recommend---just how the team do it here’. (14).*
  The ‘right timing’ was a theme that several participants mentioned as a way to avoid offence, and was related to staff awareness of spiritual needs: *‘if---it was probably pushed me on me I –I don’t think I’d feel that great. So it’s got to be appropriate. It’s hard to say-- yeah, but the timing has to be right’. (14)* The difficulty in identifying such times was acknowledged: *‘That would be the difficulty for-- to provide that, to-to not give it where it’s not needed, but to give it where it’s needed’. (16)* The diversity of expressed views demonstrated the importance of choice and agency in spiritual care delivery, as comparable with patient-centred choice and agency in medical aspects of healthcare.
  The prompts about peace were perceived as associated with end of life, as explained above, or religious in connotation, and therefore not helpful for this cohort.
- ***With an Individual Focus***

Spirituality is by definition personal and unique, and patients related best to prompts that tapped into their individual values. *‘I think each patient should be treated individually according to his or her needs. Everyone is different’. (K2)* The importance of identification of individual needs was particularly evident when rituals or religious customs needed to be observed, such as when Catholic patients desired to take daily Holy Communion *‘[be]cause you never know the minute you're going to kick the bucket’ (21)*; or Muslim patients requested halal food. As a result, the prompts about individual values were seen as preferable, such as those about what is important to the person and what keeps them strong when times are tough.

All prompts about what is important to the person were well received and seen to be helpful if considered for the individual’s care. *‘[My advice is] actually listen to the patient… what’s important to them. And if it’s having someone in the room then let-let them be in the room’. (30).*

Patients also responded well to prompts about individual sources of strength. *‘Well-being is strength… I guess” strong” is more about talking about “in the face of adversity” like this, I guess. And – -and yet having people that you love, that you can turn to is a pretty big deal’. (18).*

## Discussion

In this cross-sectional mixed-methods study, we investigated the preferences of Australian hospital patients regarding the introduction of spirituality into healthcare. We found a very high level of acceptance for enquiry about patient spirituality, although many patients felt that spiritual needs would not necessarily be present merely because of hospitalisation. The appropriateness of spiritual enquiry was seen as contextual.

While a small minority of patients in this cohort did not want to be asked about spirituality, the qualitative data suggest several reasons for this. Some patients did not see the relevance of spirituality to healthcare, some had privacy concerns and some expressed general discomfort around the topic. We collected demographic data and measures of self-assessed spirituality and religiosity for our cohort, hoping that we would identify predictors of the preference not to discuss spirituality in hospital, however the high level of acceptability of all potential spiritual history prompts meant that we were largely unable to distinguish the two groups by these variables. The significant preference for prompts about what keeps one strong when times and tough and ‘I am at peace’ by religious and spiritual participants may reflect an increased comfort with discussing existential concerns in those groups.

Our qualitative data also affirmed that spiritual needs could fluctuate, and were more likely to occur with serious illness, and also that patients may not be aware of their inability to cope prior to a crisis. These attitudes were identified in a literature review which found that patients who were not seriously ill did not want to be asked about spirituality (Best et al., 2015b). However, in the review, some individual studies found that this ‘minority’ of patients who did not want spiritual review could constitute more than 50% of patient samples.

It is therefore important that staff be sensitive to patient preferences in this area, and not insist on discussions that individual patients may not welcome. This does not include the medical history, the aim of which is to have comprehensive information about patients and their values which is required to provide appropriate care. Patients understandably may not be aware of this. Staff spiritual care training would help clinicians to introduce the topic gently, sensitively and perhaps in an educative way (Jones et al., 2021).

Some participants felt the hospital ward was insufficiently private for spiritual conversations. Privacy in hospitals is recognised as contributing to patient spiritual, as well as physical, mental and emotional well-being (Woogara, 2001) and work to improve patient privacy in hospitals is ongoing (Eijkelenboom & Blok, 2021). It is not clear whether, in this study, the participant perception of inadequate privacy applied to intimate questions on other topics as well, or whether it reflected an expectation of increased confidentiality for spiritual discussions in particular.

While principles of confidentiality have always featured in medical ethics (Beauchamp & Childress, 2019), an association with the traditional religious confessional (with overtones of inviolable confidentiality) has led to the perception for some patients that details disclosed to spiritual carers during hospitalisation would not be shared within healthcare teams (Neels, 1977). A desire to avoid disclosure was reflected in our qualitative results. Discussion on the requirements for confidentiality in spiritual care is ongoing (Carey et al., 2015). Further exploration of patients’ desire for privacy is needed to ensure that patient dignity is upheld in spiritual care.

The majority of respondents in this study were happy to discuss spirituality with members of the healthcare staff, especially when they had significant health concerns. This is consistent with recent polls demonstrating an increased interest in spirituality in Australians, which has been particularly marked since the COVID-19 pandemic (McCrindle & Renton, 2021). This trend is occurring despite the increasing secularism in Australia.

When asked about the most acceptable way to discuss spirituality, participants expressed appreciation for personalised, sensitive approaches within the therapeutic relationship. Patient desire for healthcare professionals to ask about spirituality as a way of personalising care has been previously reported (Best et al., 2014). Previous Australian studies have suggested that clinical staff prefer a one-question approach to spiritual enquiry (Best et al., 2022) and also that those more experienced in spiritual care find it most successful when they use their own words rather than a pro-forma tool (Best et al., 2015a).

Previous research has identified three levels of spiritual exploration: (1) spiritual screening, where patients are screened for spiritual concerns, or triaged; (2) spiritual history taking, where the impact of spirituality in the life of the patient is documented; and (3) spiritual assessment, where a more comprehensive examination of the spiritual needs of the patient and their spiritual resources is conducted, preferably by a spiritual care specialist, such as a chaplain (Best et al., 2020; Puchalski et al., 2009). While our aim in this paper was to identify the questions to include in a spiritual history that would be acceptable to Australian patients, we found that approaches that would be better described as ‘screening’ questions were preferred by patients.

Our study found that those prompts which focussed on individual qualities such as strength and what was important to them were most easily understood as enquiries about personal spirituality by this Australian cohort, and these are recommended for this population. In view of the frequent confusion between spirituality and religion, words with religious connotation such as ‘spirituality’ and ‘peace’ should be avoided. It is noted that the question ‘Are you at peace?’ was validated for palliative care patients (Steinhauser et al., 2006), and its use in that setting would be appropriate.

The need to avoid words with religious connotation when asking about spirituality has previously been noted in secular countries (Best et al., 2020), and also noted in previous spiritual care training in Australia (Jones et al., 2020). Future work developing education of Australian healthcare professionals in spiritual care and the development of a culturally sensitive history-taking tool for Australian healthcare should take these factors into account.

## Limitations

Non-English speaking and seriously ill patients were excluded from this study, and their needs may differ from those included. The qualitative component of this study was not intended to be generalizable and other cohorts may respond differently. Participants in this study focussed on prompts to elicit spiritual concerns, and a formal spiritual history may need a different approach. Finally, this study reported basic descriptive statistical results and supportive qualitative data as part of a mixed methods approach. More research and analysis is needed which the authors intend to pursue and present in subsequent papers.

## Conclusions

In conclusion, this study of a heterogeneous group of Australian hospital patients found that the majority of patients were happy to be asked about spirituality by clinical staff. Conversation prompts which did not have religious connotations and focussed on individual values were most effective in eliciting spiritual concerns.
